# Placental mobilization of free fatty acids contributes to altered materno-fetal transfer in obesity

**DOI:** 10.1038/s41366-021-00781-x

**Published:** 2021-02-26

**Authors:** Birgit Hirschmugl, Simone Perazzolo, Bram G. Sengers, Rohan M. Lewis, Michael Gruber, Gernot Desoye, Christian Wadsack

**Affiliations:** 1 Department of Obstetrics and Gynaecology, Medical University of Graz, Graz, Austria; 2 BioTechMed-Graz, Graz, Austria; 3 School of Engineering, Bioengineering Research Group, University of Southampton, Southampton, UK; 4 Institute for Life Sciences Southampton, University of Southampton, Southampton, UK; 5 University of Southampton, Faculty of Medicine, Southampton General Hospital, Southampton, UK

**Keywords:** Obesity, Fatty acids

## Abstract

**Background:**

Metabolic changes in obese pregnant women, such as changes of plasma lipids beyond physiological levels, may subsequently affect fetal development in utero. These metabolic derangements may remain in the offspring and continue throughout life. The placenta mediates bidirectional exchange of nutrients between mother and fetus. The impact of prepregnancy obesity on placental transfer of lipids is still unknown.

**Objective:**

We aimed to examine materno-to-fetal free fatty acid (FFA) transfer by a combined experimental and modeling approach. Flux of ^13^C-labeled FFA was evaluated by ex vivo perfusion of human placentae as a function of prepregnancy obesity. Mathematical modeling complemented ex vivo results by providing FFA kinetic parameters.

**Results:**

Obesity was strongly associated with elevated materno-to-fetal transfer of applied ^13^C-FFA. Clearance of polyunsaturated ^13^C-docosahexaenoic acid (DHA) was most prominently affected. The use of the mathematical model revealed a lower tissue storage capacity for DHA in obese compared with lean placentae.

**Conclusion:**

Besides direct materno-to-fetal FFA transfer, placental mobilization accounts for the fetal FA supply. Together, with metabolic changes in the mother and an elevated materno-fetal FFA transfer shown in obesity, these changes suggest that they may be transmitted to the fetus, with yet unknown consequences.

## Introduction

Obesity has become a global burden as the prevalence has continuously increased in the last decades. Consequently, increasing numbers of women in the reproductive age are also affected by this disease. Obesity is defined by an excess of body weight for height and is expressed by a body mass index (BMI) score of >30 kg/m^2^ [1]. Obesity and pregnancy are linked with a number of pregnancy complications for the mother. In particular, obese mothers are at higher risk of developing hypertension, preeclampsia, and gestational diabetes [2]. Maternal obesity also increases risk for a delayed labor onset and slow labor progress that often results in unplanned cesarean delivery [3]. Prepregnancy obesity can affect the growth of the fetus in utero. Notably, elevated percentage of neonatal body fat and concurrently normal birth weight were reported [4]. Infants of obese mothers are at a higher risk to be born large for gestational age [5, 6] and subsequently develop childhood obesity [6, 7], a predictor of adulthood obesity [8]. Thus, infants of obese women are at a high risk of entering into the generational spanning obesity–diabetes cycle.

Facing childhood obesity as a serious public health problem, it would be necessary to disclose the origin of fetal overgrowth and understand the mechanisms resulting in childhood obesity.

In this context, some studies report alterations in maternal plasma lipid profile in obese women such as elevated triglyceride levels [9, 10], or increased insulin resistance [11–13] compared to lean pregnant women. However, the immediate contribution of maternally derived metabolic changes to excessive lipid accumulation in fetal adipose tissue is still elusive. All exchange processes between the maternal and fetal circulation are mediated by the placenta, and therefore appropriate placental function is crucial for fetal development. The fetus is dependent on the supply of maternal long-chain polyunsaturated fatty acids (LC-PUFA) that are specifically required for an appropriate development of the brain and neuronal system [14]. Recent studies have shown that metabolic changes, as a result of obesity even before pregnancy, are associated with elevated placental lipid accumulation [15–17] and impaired mitochondrial function that is further related to increased oxidative stress in placental tissue [16, 18]. Alterations in placental expression of fatty acid transport proteins (FATP), fatty acid translocase (CD36), or fatty acid binding proteins (FABP) were reported in relation to obese conditions in mice and humans [9, 19, 20]. FATP and CD36 are membrane proteins which are involved in the cellular uptake of free fatty acids (FFA). Once FFA are taken up by the placenta, they are bound to FABP and further processed to membrane phospholipids, used for ATP production in mitochondria, or esterified to triacylglycerols as the intermediate storage form in cells [21]. Although lack of direct evidences, maternal obesity alters at least placental lipid homeostasis which eventually may alter lipid supply to the fetus.

The driving mechanisms underlying the transplacental FFA transfer need to be elucidated. The question arises if and how the placenta is affected and regulated under pathophysiological metabolic conditions, such as maternal obesity. In this study with a sole focus on the placenta, we aimed to examine alterations in placental FFA transfer in respect to maternal prepregnancy obesity. Therefore, we used the ex vivo dual placental perfusion approach to study direct transfer of ^13^C-labeled FFA across placental tissue of obese and lean mothers. In addition, the mathematical model for placental FFA transfer mechanisms, previously published by Perazzolo et al. [22] was applied to elucidate further key factors underlying the obtained experimental materno-to-fetal FFA transfer results in placentas of obese compared to lean mothers.

## Materials and methods

### Study subjects

Women either with a prepregnancy body mass index (BMI) < 25 kg/m² (lean, *n* = 8) or BMI ≥ 30 kg/m² (obese, *n* = 7) were included in this study. Gestational age of all women was >38 weeks and all delivered by elective cesarean section. According to the medical records, participating women were fasted overnight and did not take any medication. All women were normotensive. Gestational diabetes was excluded by oral glucose tolerance test between gestational week 24 and 28. The study followed the declaration of Helsinki was approved by the ethics committee of the Medical University of Graz (EK No. 24-529 ex 11/12) and written informed consent was signed by all study participants.

### Reagents and equipment

For all placental perfusion experiments Dulbecco´s modified eagle medium (DMEM, phenol red free, Gibco, UK) was used. DMEM was mixed with Earl´s buffer (6.8 g/L NaCl, 0.4 g/L KCl, 0.14 g/L NaH_2_PO_4_, 0.2 g/L MgSO_4_•7 H_2_O, 0.2 g/L CaCl_2_, 2.2 g/L NaHCO_3_, all Merck, Darmstadt, Germany) in a 3:1 medium buffer ratio containing amoxicillin (250 mg/L, Sigma-Aldrich, Steinheim, Germany), dextran FP40 (10 g/L, Serva, Heidelberg, Germany), 2 g/L glucose (Merck, Darmstadt, Germany), and essential fatty acid free bovine serum albumin (BSA) (5 g/L, Sigma-Aldrich, Seinheim, Germany).

Perfusion medium containing ^13^C-labeled free fatty acid mix (^13^C-FFA mix) was prepared under a stream of argon to prevent fatty acid oxidation. The albumin to FFA molar ratio was 0.77, which is in the physiological range in the systemic circulation of a pregnant women [9, 23]. Preparation details and FFA final concentrations are shown in Supplementary Table 1.

### Ex vivo dual placental perfusion setup

The ex vivo placental perfusion set up used in this study was adapted and technically extended based on the approach of Schneider et al. [24]. Briefly, within 30 min after delivery of the placenta a corresponding chorionic artery and vein pair supplying one intact cotyledon was cannulated. The cannulated cotyledon was immediately flushed with pre-warmed (37 °C) perfusion medium, thereafter this cotyledon and surrounding tissue was placed in the pre-warmed perfusion chamber. Finally, the cannula of the fetal artery was connected to the reservoir containing perfusion medium kept at 37 °C in a water bath. Gas levels in the medium were adapted by a gas exchange device (Living Systems, St. Albans, VT, US) operated with 95% N_2_ and 5% CO_2_ during the experiment. The fetal circulation was always perfused with fresh medium (open circulation), which allowed us to distinctly follow each FA released from the placenta. A constant fetal artery inflow of 4 mL/min was set up by using a magnetic pump (Codan, Salzburg, Austria). Volume loss was monitored within the first 30 min, thereafter every hour at the fetal venous outflow port. Each cotyledon displayed at least 95% fetal flow recovery. The backflow pressure in the fetal vascular system was recorded by a micro catheter pressure sensor (Millar, US) inserted into the fetal arterial cannula. Experiments with vessel back pressure lower than 65 mbar were considered as successful [25].

Next, maternal circulation was set up by inserting three rounded needles into the intervillous space of the cotyledon. During the experiment, medium in maternal circulation was gassed with 5% CO_2_, 20% O_2,_ and 75% N_2_ by applying a gas exchange device. The maternal flow rate was kept at 8 mL/min. Within the first phase of perfusion experiment, antipyrine (100 µg/mL, Sigma-Aldrich, Schnelldorf, Germany) was added to the maternal reservoir and perfusion was operated by open circulations. Antipyrine crosses the placental barrier by passive diffusion and serves as quality control substance for the size of the materno-to-fetal exchange area [26].

Samples from maternal artery and fetal vein outflow (perfusates) at the time points 0, 10, 20, and 30 min were collected, centrifuged at 3000 rpm at 4 °C for 10 min, and stored at −80 °C until antipyrine concentrations were determined.

Thereafter, the maternal reservoir was switched to perfusion medium containing ^13^C-FFA mix (200 mL, 0.5% BSA) and the system changed to closed circuit at the maternal side. Samples of both circuits were collected at 0, 10, 20, 30, 60, and 90 min, centrifuged and stored at −80 °C. Finally, maternal reservoir was switched to media containing 0.5% BSA with an open circuit configuration for a further 30 min. At the end of the experiment, wet weight of the perfused cotyledon was determined and is designated as perfused tissue mass (ptm) in the calculations and statistics section.

In order to assess viability of the perfused cotyledon, maternal and fetal perfusates were sampled (1.5 mL, via sampling port) during the experiment every 30 min. Oxygen (pO_2_), carbon dioxide (pCO_2_), pH, lactate production, and glucose consumption were measured by a blood gas analyzer (Radiometer, Copenhagen, Denmark). LabVIEW based recording software (Beko engineering, Graz, Austria) was used to register data sets obtained by the blood gas analyzer, the magnetic pumps, and the pressure sensor.

### Antipyrine quantification by HPLC

Antipyrine concentrations of maternal and fetal perfusates were determined by HPLC as described by Annola et al. [27]. Briefly, 100 µL of perfusate was mixed with 100 µL methanol (Sigma-Aldrich, Schnelldorf, Germany), vortexed and centrifuged at 12000 rpm for 15 min. Upper phase (150 µL) was transferred to a new tube and mixed with 150 µL acetonitrile (Sigma-Aldrich, Schnelldorf, Germany), again vortexed and centrifuged. A series of standards with antipyrine concentrations from 5 µmol/L to 1 mmol/L was prepared as described above. Antipyrine concentrations were determined by HPLC (Knauer, Berlin, Germany), which was equipped with an aquasil 150 × 2.15 µ column (Thermo scientific, Waltham, MA, USA) and an UV detector (Knauer, Berlin, Germany). Either 10 µL sample or standard was injected, followed by an isocratic flow (0.2 mL/min) run with 20 mmol/L KH_2_PO_4_ (Merck, Darmstadt, Germany) in water mixed 1:1 with acetonitrile. Antipyrine peaks were detected at 255 nm and antipyrine concentrations were calculated according to the obtained standard curve.

Antipyrine fetal/materno ratio (FM ratio) was calculated and only perfusion experiments with a FM ratio ≥0.3 in 30 min were considered as a successful experiment and further processed.

### Fatty acid analysis by gas chromatography–mass spectrometry (GC–MS)

Total lipids, out of 1 mL perfusion medium, were extracted in methanol/tert-butyl methyl ether/water (1.5/5/1.25) as described by Matyash et al. [28]. Samples were spiked with C15:0 FFA (3.75 nmol/mL) as internal standard. The organic phase was dried in a vacuum centrifuge. Lipids were dissolved in 500 µL CHCl_3_/methanol (1/1). FFA were determined out of total lipid extracts, as previously published by Fuchs et al. [29] with some modifications. Briefly, solvent of lipid extracts (200 µL) were evaporated, resolved in 50 µL of a pentafluorobenzyl bromide solution (3.4% in acetonitrile), and 10 µL of N, N-diisopropyl ethanolamine. Samples were incubated at room temperature for 10 min, evaporated under a gentle stream of nitrogen, finally resuspended in 50 µL of hexane and used for FFA analysis.

All ^13^C-labeled FFA carried ^13^C isotopes on each C-atom position. The labeling efficiencies were 97% atoms for DHA and 99% atoms for the other FFA, as specified by the manufacturers. In order to verify retention time and mass of the individual ^13^C-FFA, we performed a trial measurement of each single ^13^C-FFA by GC–MS, equipped with a quadrupole triple-axis detector (Agilent Technologies, Santa Clara, CA, USA), run on negative ion chemical ionization and selected-ion monitoring (SIM) mode (a list of masses given in Supplementary Table 1). FFA species, including ^13^C-labeled FFA were detected by retention time (according to mixed FFA standards) and mass, after loss (m-1) in SIM scan mode. To quantify specific FFA, areas under the curve (AUC) were calculated by Mass Hunter (Agilent Technologies, Santa Clara, CA, USA). Ratios between internal standard (3.75 nmol/mL C15:0 FFA) and each individual FFA species were calculated. Additionally, FFA in perfusates containing 0.5% BSA (blank, without ^13^C-FFA mix) were determined, and obtained values (mainly low levels of 16:0 and 18:0) were subtracted from measured FFA concentration of each sample. No corrections were made for natural abundance of ^13^C since each C-atom carried an ^13^C-isotope of used labeled FFA and a defined mass shift depending on FFA chain length of +16 (palmitic acid), +18 (oleic and linoleic acid), or +22 (DHA), respectively. We assumed equal response factors for all labeled and un-labeled FFA. All reagents were purchased form Sigma-Aldrich, Steinheim, Germany, if not other stated.

### Mathematical modeling

The mathematical model used here has been described in detail previously [22]. Briefly, a compartmental modeling approach was adopted to predict the concentrations involved in the experimental setup: maternal artery, maternal vein, syncytiotrophoblast, and fetal vein. All compartments were assumed to be well-mixed, while transfer between compartments was described by facilitated diffusion. In this study, the model was used specifically to represent the ^13^C-DHA uptake and transfer in comparison with the experimental data. By fitting the model to the data, four parameters were estimated: the maximum uptake flux parameter for the microvillous membrane (v_MVM_), the maximum delivery flux parameter for the basal membrane (v_BM_), the syncytiotrophoblast metabolic accumulation rate constant (k_acc_) and the syncytiotrophoblast metabolic release rate constant (k_rel_). The parameters were determined separately for each of the lean and obese subjects, averaged for each group and tested for significant difference between groups. To test the predictive capabilities of the model, an additional independent experiment was performed. The first phase of the experiment was performed with ^13^C-FFA mix containing 0.3 µmol/L ^13^C-DHA (see also Supplementary Table 1), after 120 min, the experiment was continued by switching back to closed loop perfusion with a new reservoir concentration of 1.5 µmol/L ^13^C-DHA in the FFA mix (five times the dose given at time zero). The parameters extracted from the first phase of the experiment (0–120 min) were then used for the prediction of the second phase from 120 min to 210 min.

### Calculations and statistics

Antipyrine transfer was calculated as followed.

$$FM\,ratio = \frac{{fv}}{{ma}}$$

fv: concentration in fetal vein; ma: concentration in maternal artery

Materno-to-fetal FFA transfer was normalized to mean perfused tissue mass (25 g, Supplementary Table 2), and calculated as clearance.

$$Clearance = \frac{{\frac{{fv}}{{ptm}} \ast 25}}{{ma}} \ast Qf$$

fv: concentration in fetal vein; Qf: fetal flow rate (mL/min); ma: concentration in maternal artery; ptm: perfused tissue mass (g)

Isotopic enrichment (%) was calculated as followed.

$$Isotopic\,enrichment\,\left( {\mathrm{\% }} \right) = \frac{{fv}}{{(fv + fv\,pl)}} \ast 100$$

fv: ^13^C-FFA concentration in fetal vein; fv pl: placental released non-labeled FFA concentration in fetal vein

^13^C-FFA tissue uptake (%) was calculated as followed.

$$Uptake\left( {\mathrm{\% }} \right) = \frac{{\left( {mr\,t0 - mr\,t90 - fr\,t90} \right)}}{{mr\,t0}} \ast 100$$

mr t0: ^13^C-FFA absolute quantity in the maternal reservoir at time point 0 min; mr t90: ^13^C-FFA absolute quantity in the maternal reservoir at time point 90 min; fr t90: ^13^C-FFA absolute quantity in the fetal reservoir at time point 90 min

Data are presented as mean ± SD. Statistical analysis was performed by two-way ANOVA with Bonferroni post hoc test (^13^C-FFA clearance), non-parametric group comparison Mann–Whitney *U* test (model parameter for ^13^C-FFA and study population characteristics) and Kruskal–Wallis with post hoc Dunn´s test (FFA placental release in comparison to ^13^C-FFA transfer). *P* values <0.05 were considered significant. For statistical analysis GraphPad Prism 7.0 (GraphPad Software, La Jolla, CA) and IBM SPSS Statistics (version 23) was used.

## Results

### Evaluation of materno-to-fetal FFA transfer by combining experimental and mathematical model approaches

**Fig. 1:**
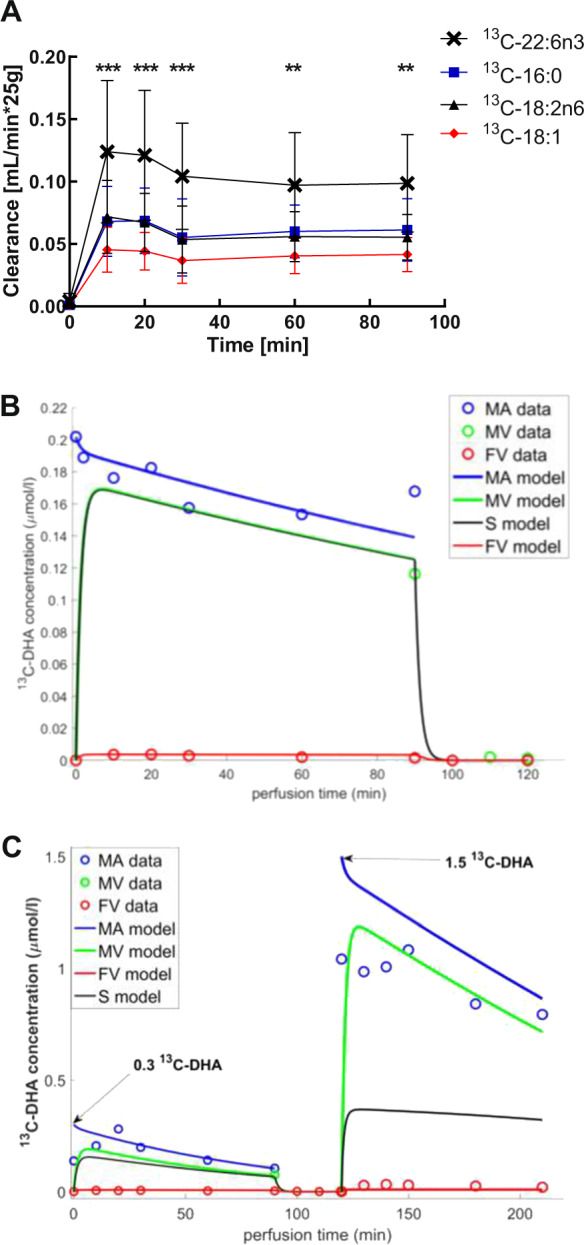
Clearance of ^13^C-labeled FFA and model prediction fitting the perfusion data for the ^13^C-DHA placental transfer. (**A**) Clearance of ^13^C-labeled palmitic acid (16:0), oleic acid (18:1), linoleic acid (18:2n6), and docosahexaenoic acid (22:6n3) was examined by placental perfusion (*n* = 13). Kinetic of FFA transfer was investigated utilizing a closed (recirculated) maternal and open (nonrecirculating) fetal circulation. Mean (± SD) ^13^C-FFA clearance was calculated at time points 0, 10, 20, 30, 60, and 90 min. Two-way ANOVA and Bonferroni post hoc test were performed, ** p < 0.01and *** p < 0.001 indicate significant differences between ^13^C-DHA and other ^13^C-FFA. (**B**) Circles represent the experimental data, solid lines represent the model predictions for the ^13^C-DHA concentration of one lean subject. Blue represents the maternal artery (MA); green represents the maternal vein (MV); black represents the syncytiotrophoblast compartment (S); red represents the fetal vein (FV). Note that maternal vein concentrations were measured only at t = 90 min, whilst no experimental data were available for the tissue concentrations. Model parameter from one representative experiment is illustrated. (**C**) Model prediction when 0.3 µmoL/L ^13^C-DHA was added at time zero, followed by 1.5 µmoL/L at t = 120 min. The total perfusion time was 210 min. Circles represent the experimental data, solid lines for the model predictions. Blue represents the maternal artery (MA); green represents the maternal vein (MV, t = 90 min only); black represents the syncytiotrophoblast compartment (S, no experimental data available); red represents the fetal vein (FV). The model parameters were estimated solely based on the data from the first phase of the perfusion experiment (0–120 min) and then applied to predict the second phase of the experiment (120–210 min). Note that the initial concentration values used for the model were based on the initial dose administered. This particular experiment was performed once.

The placental transfer for palmitic acid (PA, 16:0), oleic acid (OA, 18:1), linoleic acid (LA, 18:2n6), and docosahexaenoic acid (DHA, 22:6n3) was examined by placental ex vivo perfusion. Stable isotope ^13^C-labeled PA, OA, LA, and DHA together with a combination of non-labeled FFA were applied to perfusion medium containing albumin. All ^13^C-labeled FFA were already detectable in fetal effluent after 10 min perfusion (Fig. 1A). The long chain polyunsaturated ^13^C-DHA clearance was significantly more pronounced at all measured time points than the clearance for ^13^C-PA, ^13^C-LA, and ^13^C-OA.

Therefore, we implemented ^13^C-DHA transfer data in our previously published mathematical model [22]. The mathematical model was able to effectively match the current experimental data for ^13^C-DHA presented in one representative plot (Fig. 1B). A robust model must be able to work under a range of different conditions. In order to test the reliability of the mathematical model, the perfusion protocol was adapted and the experiment extended with a second higher dose of ^13^C-DHA (1.5 µmol/L; Fig. 1C). The correspondence between model and experiment demonstrated how the model was able to predict reasonably well-perfusion experiments for different ^13^C-DHA input conditions based on the kinetics estimated during the first phase of the experiment. Note that due to the experimental variation in this particular case the initial concentrations used in the model were based on the initial dose administered, which is higher than the first concentrations measured in the experiment. The kinetic model parameters estimated for ^13^C-FFA in the lean and obese group are presented in Table 1. For each examined ^13^C-FFA (PA, OA, LA, and DHA), the v_MVM_ was significantly (*p* < 0.01) higher than the v_BM_ by over two orders of magnitude, implying that the effective permeability of the MVM was much greater than from the BM. Comparing the lean control and obese group there was a trend for a difference in the accumulation into metabolism parameter k_acc_ for ^13^C-DHA, but it was not significant (*p* = 0.08). The estimated parameter k_rel_ for metabolic release of ^13^C-FFA during the experiment was zero in both cases meaning no relevant release back in the system after sequestration by the metabolic pool in the 90 min experimental period.

**Table 1:** Estimated model parameters for ^13^C-FFA of lean and obese experiments.

	v_MVM_ (µmol/min)		v_BM_ (µmol/min)		k_aac_ (1/min)	
^13^C-16:0						
Lean	17.1	(± 14.9)	0.008	(± 0.006)	0.65	(± 0.58)
Obese	18.5	(± 26.2)	0.014	(± 0.008)	1.58	(± 2.28)
^13^C-18:1						
Lean	25.5	(± 18.5)	0.132	(± 0.340)	0.54	(± 0.77)
Obese	27.9	(± 51.8)	0.012	(± 0.006)	0.73	(± 1.26)
^13^C-18:2n6						
Lean	16.0	(± 23.3)	0.044	(± 0.107)	0.59	(± 1.05)
Obese	11.9	(± 19.0)	0.005	(± 0.004)	0.34	(± 0.39)
^13^C-22:6n3						
Lean	0.083	(± 0.059)	0.00004	(± 0.00003)	0.86	(± 0.96)
Obese	0.124	(± 0.113)	0.00004	(± 0.00002)	0.34	(± 0.17)

v_MVM_ represents the maximum uptake flux parameter for the MVM (µmol/min); v_BM_ is the maximum flux parameter for the BM (µmol/min); k_acc_ depicts the metabolic accumulation rate constant (1/min). The rate constant k_rel_ for metabolic release of labeled fatty acid from the placental tissue is equal to zero and not reported in the table. Mean (± SD) of lean control (*n* = 8) and obese (*n* = 7) groups are shown. There are no statistically significant differences between control and obese groups but there was a trend for a difference in ^13^C-22:6n3 k_acc_ (*p* = 0.08). Non-parametric group comparison (Mann–Whitney *U* test) was performed.

Reliability of obtained ex vivo perfusion experiments is shown by monitoring specific metabolic parameters in accordance with recommended practice [25] (Supplementary Table 2).

### Effect of maternal prepregnancy obesity on trans-placental FFA transfer

Study population (*n* = 15) was classified according to maternal prepregnancy BMI into a lean (BMI 18.5–24.9 kg/m², *n* = 8) and obese group (BMI ≥ 30 kg/m², *n* = 7). The obese group was matched for maternal age and gestational age. Neither placental weight nor birth weight or ponderal index was significantly different between lean and obese groups (Supplementary Table 3).

**Fig. 2:**
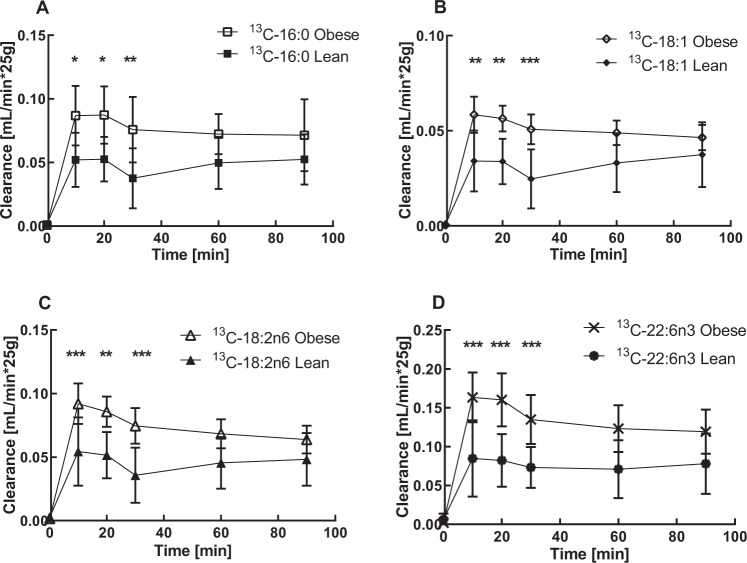
Clearance of ^13^C-labeled FFA in placentae of lean versus obese women. Direct transfer of ^13^C-labeled **A** palmitic acid (16:0), **B** oleic acid (18:1), **C** linoleic acid (18:2n6), and **D** docosahexaenoic acid (22:6n3) was determined in lean (*n* = 7) and obese (*n* = 6) placentae by ex vivo perfusion. Kinetic of FFA transfer was investigated utilizing a closed (recirculated) maternal and open (nonrecirculating) fetal circulation. Mean (± SD) ^13^C-FFA clearance was calculated at time points 0, 10, 20, 30, 60, and 90 min. Two-way ANOVA and Bonferroni post hoc test were performed, one experiment in each group was excluded from the analysis due to missing FFA measurements at one single time point, * *p* < 0.05, ** *p* < 0.01, and *** *p* < 0.001.

The impact of maternal prepregnancy obesity on FFA transfer to the fetus in late pregnancy was investigated by comparing placental clearance of ^13^C-PA, ^13^C-OA, ^13^C-LA, and ^13^C-DHA of lean (*n* = 7) and obese (*n* = 6) women (one experiment in each group was excluded from statistical analysis due to missing FFA measurement at one single time point). Independent of the group, clearance for all investigated ^13^C-FFA occurred within the first 10 min, with a maximum between 10 and 20 min and an equilibrated ^13^C-FFA-clearance after 30–90 min of perfusion. A significant increase in ^13^C-FFA-clearance across the placental barrier was observed in the obese group (Fig. 2A–D). Within both the lean and obese groups, clearance followed the order DHA > PA = LA > OA (0.082, 0.053, 0.051, 0.034 mL/min and 0.16, 0.087, 0.086, 0.057 mL/min) after 20 min, respectively (Fig. 2A–D).

We determined the placental release of endogenous FFA, which were present in placental tissue before the experiment, and the concentration of ^13^C-FFA in fetal perfusates after 90 min. For these calculations we had to exclude two experiments in the lean and one experiment in the obese group due to 3-fold higher ^13^C-DHA concentration in maternal perfusates at the beginning of the experiment. The placental release of endogenous PA, OA, and LA tend to be lower in placentae of obese compared to lean mothers, notably the endogenous DHA release was significantly lower in the obese group (*p* = 0.04, Table 2). The concentration of all applied ^13^C-FFA was elevated in fetal perfusates of obese compared to lean placentae. Interestingly, only ^13^C-PA levels were significantly elevated in obesity.

**Table 2:** Release of endogenous and ^13^C-labeled FFA to the fetal circulation.

	16:0		18:1n9		18:2n6		22:6n3	
Endogenous FFA								
Lean	0.642	(± 0.222)	0.263	(± 0.098)	0.378	(± 0.179)	0.072	(± 0.030)
Obese	0.627	(± 0.295)	0.203	(± 0.037)	0.3	(± 0.066)	0.045*	(± 0.013)
^13^C-FFA								
Lean	0.118	(± 0.051)	0.157	(± 0.056)	0.085	(± 0.028)	0.001	(± 0.001)
Obese	0.195*	(± 0.055)	0.187	(± 0.057)	0.108	(± 0.033)	0.002	(± 0.001)

Endogenous FFA and ^13^C-labeled FFA were determined in fetal perfusates after 90 min. Results are adjusted to average perfused cotyledon mass (25 g) and are expressed as mean (± SD) µmol/90 min of perfused lean (*n* = 6) and obese (*n* = 6) placentae. Differences in endogenous FFA and ^13^C-FFA were tested between lean and obese placentae (Mann–Whitney *U* test, * *p* < 0.05).

**Fig. 3:**
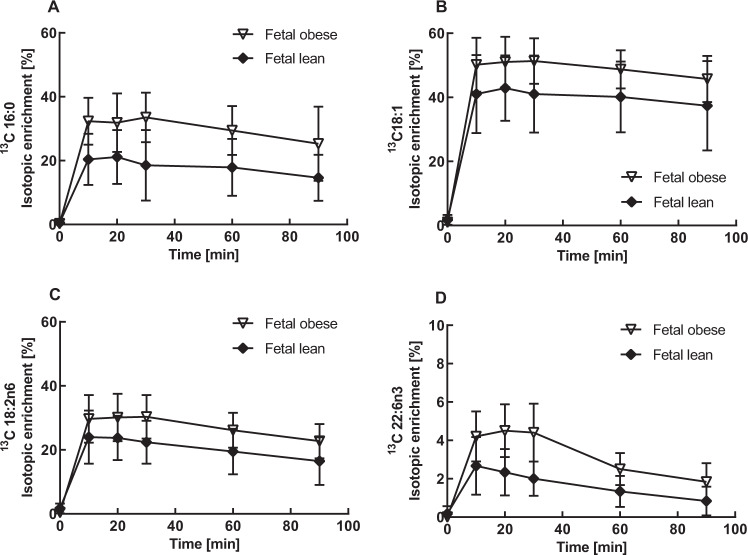
Isotopic enrichment in fetal effluents. ^13^C-labeled and endogenous FFA were determined in fetal venous samples **A** palmitic acid (16:0), **B** oleic acid (18:1), **C** linoleic acid (18:2n6), and **D** docosahexaenoic acid (22:6n3). Mean (± SD) ^13^C-isotopic enrichment in perfusion experiments, lean (*n* = 8) and obese (*n* = 7) placentae were calculated at time points 0, 10, 20, 30, 60, and 90 min and is expressed as percentage.

Next, we calculated the isotopic enrichment of ^13^C-labeled FFA over time in order to state the mobilization kinetic for placental endogenous FFA. The isotopic enrichment in the fetal venous perfusates of ^13^C-labeled FFA was constant over time but varied between the examined FFA. We found the highest enrichment for ^13^C-OA (40–55%) followed by ^13^C-PA, ^13^C-LA (20–35%) and lowest enrichment for ^13^C-DHA with 3–5% (Fig. 3). Furthermore, the isotopic enrichment for all ^13^C-FFA was higher in obese compared with lean placentae.

No uptake differences of offered ^13^C-FFA into the placental metabolic pool between lean and obese women could be observed (Supplementary Fig. 1).

## Discussion

During intrauterine life a proper development of the fetus depends on maternally derived polyunsaturated FFA. Prior studies have noted a direct link between maternal intake of LC-PUFA with growth measures at birth and development of the fetal brain [30, 31]. The placenta coordinates the preferential uptake and transfer of LC-PUFA from the maternal to the fetal circulation, but how the tissue balances this trafficking is still poorly understood. Moreover, most of the studies in this field ignore the capability of the placenta itself to store, metabolize, and to release FAs for different needs. Therefore, an initial objective of this study was to identify how normal placentae compared to placentae from prepregnant obese women differ in respect to FA handling.

The present study is the first to address holistically materno-to-fetal FFA transfer, including to study endogenously provided FFA of the human placenta. We approached this by using transfer data of ^13^C-labeled FFA and applied the results to the recently published mathematical model [22]. In addition, the model approach was extended by DHA, which represents as one of the most important unsaturated FA for fetal development. The strength of the used experimental approach is that it enables simultaneous analysis of transferred labeled FFA and released (endogenous) FFA from the tissue to the fetal circulation. In general, placentae from obese subjects showed a higher clearance for all applied FFA compared to lean in the initial phase of tissue perfusion. More specifically, clearance of DHA was two times higher in obese placentae than in lean, suggesting a preferred role of DHA for these specific conditions. However, in general, clearance of all applied FA is low compared to the contribution of tissue released FA. Independently, the general very low clearance of all applied FA emphasizes the importance of lipid accumulation and an altered metabolism in the tissue. In line, it has been shown that maternal BMI is linked to an altered expression of lipid transporters and fatty acid composition in the tissue [32]. The existence of placental intracellular lipid stores has already been shown by us and others [16, 17, 33]. In particular, isolated placental trophoblasts accumulate lipids actively after incubating with FA, a mechanism which is enhanced under pro-inflammatory conditions. Whether an altered cellular FA uptake or metabolic changes may account for these differences cannot be ultimately resolved by using static cell culture model system [34]. One unanticipated finding was that under physiological flow conditions, non-labeled FFA appear in the fetal circulation in the same time. It is very likely that placental tissue mobilizes endogenous FFA from intracellular lipid stores in order to cover fetal demand under certain metabolic conditions. Release of non-labeled FFA to the fetal compartment exceed 2–3 times of ^13^C-transferred FA, except for DHA. In the fetal circulation mobilized and released DHA from the placenta exceeds many times over transferred ^13^C-DHA, which was maternally offered in a physiological range (Table 2).

In this study we demonstrate that direct transfer of saturated and unsaturated FFA from the mother to the fetus is elevated in obese compared to lean women. However, maximum membrane flux parameters at the MVM and the BM obtained from in silico evaluation were similar between lean and obese placentae, suggesting that other variables than membrane fluxes may account for this effect. Interestingly, the rate constant for the metabolic DHA-accumulation in the model (k_acc_) was lower in obese compared to lean placentae, indicating a favorable direct transfer of DHA to the fetal circulation under this specific metabolic condition, which matches our experimental data.

Recently, Gazquez et al. published two in vivo tracer studies where lean and obese women received ^13^C-FFA orally prior to cesarean section [35, 36]. Although these and our studies touching a similar area, a direct comparison of absolute FA-levels or ratio of ^13^C-FA enrichment in the placenta is inaccurate, because of different timing for collected samples. First, labeled FFA were administered orally 12 h before delivery. Second, while cord blood was taken directly after birth, last maternal blood sample was taken 2 h before delivery which cannot be defined as matched samples. In contrast, our experimental setting enabled to collect maternal and fetal samples simultaneously at distinct time points after administrating ^13^C-FA. Interestingly, in both studies compared to lean placentae, the trend for lower DHA levels in placenta and cord blood affected by obesity is identifiable. In our study decreased levels of endogenous DHA were seen in obesity, and weighs heavier than the transferred ^13^C-DHA (Table 2).

The other examined FA, ^13^C-stearic acid, ^13^C-palmitic acid, and ^13^C-oleic acid, showed a materno-fetal transfer of around ~1% [35]. These values are comparable to our results obtained for palmitic acid by ex vivo perfusion approach. However, the transfer kinetics for oleic acid were slightly lower and that of DHA, which was significantly higher by our ex vivo approach. That was previously also observed by Haggarty et al. [37], and raised a concept of FA-transfer across the placenta that involves fatty acid transport proteins (FATP) and fatty acid binding proteins (FABP) [21].

Our approach using the ex vivo placental perfusion technique in combination with in silico modeling highlights the importance of including the endogenous capability of the placenta to mobilize and release (unsaturated) FA, beside materno-to-fetal transfer, which overall cannot be fully accessed by in vivo studies. In our in silico model ^13^C-FFA transfer to the fetal reservoir and accumulation in the placental lipid pool was addressed, but there was no relevant ^13^C-FFA release from the placental lipid pool. Since the uptake parameter exceeds the release parameter by over two orders of magnitude, remobilization of ^13^C-FFA may take longer than 90 min. A limitation of the in silico model is that it could not cover mobilization and release of endogenous FFA present in placental tissue before the experiment, as we observed it in the ex vivo experiments.

The low number of investigated subjects is certainly a methodological limitation of our study. Nevertheless, in comparison to other ex vivo perfusion studies, enrolled tissue perfusions are of high experimental quality [25] and the number of investigated placentas are comparative [38]. Unfortunately, we could not assess metabolic and inflammatory parameters of obese women. However, as we know from other studies, surrogate clinical parameters like weight, height of the offspring, or placental morphology might not reflect its metabolic condition [39, 40]. It is possible that the increased FFA flux across obese placentae contributes to the elevated lipid accumulation in fetal adipose tissue and therefore altering body composition rather than birth weight [4].

**Fig. 4:**
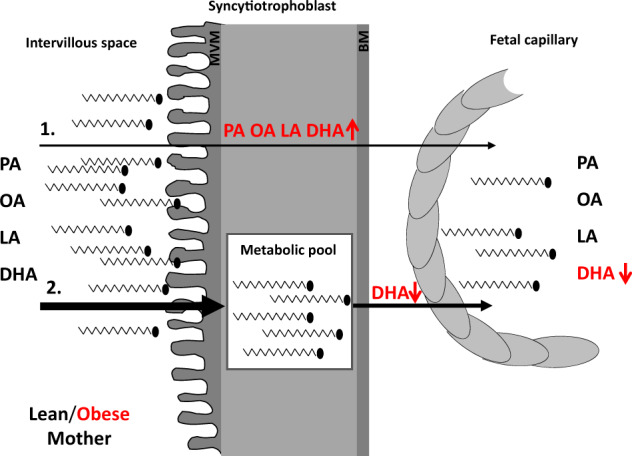
Model for FFA transfer across the placental barrier. (**1**) FFA derived from maternal plasma is transferred directly across the placental syncytiotrophoblast to the placental–fetal endothelium. In placentae of obese women transfer rates for all examined FFA are elevated (red arrow). (**2**) Simultaneously, maternal FFA are taken up by the placenta and mass balance calculations suggest an enrichment of FFA within the tissue (metabolic pool). Out of the metabolic pool, FFA may be released to the fetal circulation. In fact, mobilization of DHA was reduced in placentae of obese women, resulting in lower DHA levels in the fetal capillaries/circulation. MVM microvillous membrane, BM basal membrane, PA palmitic acid, DHA docosahexaenoic acid, LA linoleic acid, and OA oleic acid.

To conclude, the importance of this area is illustrated by our findings in which only a small proportion of FFA are directly transferred across the placenta in comparison to the FFA proportion released by the metabolic pool in the same time. The exogenous FFA transfer capacity in the syncytium reaches a maximum in a short time period implying that this process contributes only slightly to the materno-to-fetal FA supply. In contrast, tissue stored FA (mainly in phospholipids) are an efficiently and quickly available source of FA for the release to the fetus. Overall, our data suggest an expanded compartment mediated concept of FA uptake, storage, and delivery to the fetus in the human placenta (Fig. 4). Specific placental metabolic alterations seem to contribute more to the unfavorable newborn outcome than thought before, and further prime the offspring towards the generation spanning obesity cycle [41]. Breaking the cycle of intergenerational obesity needs more prospectively planned cohort studies. Together, our applied combined in silico and ex vivo approach presented in this study provides a comprehensive integrated approach to study materno-to-fetal lipid trafficking in normal but, more importantly, in pathophysiological pregnancies.

## Supplementary information

Supplemental Data
