# Supplementary material for: Placental mobilization of free fatty acids contributes to altered materno-fetal transfer in obesity
Source: Int J Obes (Lond). 2021 Feb 26;45(5):1114–23. doi: 10.1038/s41366-021-00781-x (PMC8081658; doi:10.1038/s41366-021-00781-x)
Supplement: Supplementary file 1 — Supplemental Data [file 41366_2021_781_MOESM1_ESM.pdf]

Supplementary Data

Placental mobilization of free fatty acids contributes to altered materno-fetal transfer in obesity

Birgit Hirschmugl[1,2], Simone Perazzolo[3,5], Bram G Sengers[3,5], Rohan M Lewis[4,5], Michael Gruber[1], Gernot Desoye[1], Christian Wadsack[1,2#]

[1]Department of Obstetrics and Gynaecology, Medical University of Graz, 8036 Graz, Austria.

[2]BioTechMed-Graz, Austria.

[3]School of Engineering, Bioengineering Research Group, University of Southampton, SO17 1BJ Southampton, UK.

[4]University of Southampton, Faculty of Medicine, Southampton General Hospital, SO16 6YD, Southampton, UK.

[5]Institute for Life Sciences Southampton, University of Southampton, SO17 1BJ, UK.

[#]Correspondence: Christian Wadsack, PhD; Medical University of Graz, Austria; Department of Obstetrics and Gynaecology; christian.wadsack@medunigraz.at

Supplementary Table 1: Composition and determined concentrations of the FFA mix

**Fatty acid**		**Concentration µmoL/L (± SD)**		**SIM Mass (m-1)**
**^{13}C-PA**	palmitic acid (16:0)	38.79	(± 13.32)	271.5
**^{13}C-OA**	oleic acid (18:1n9)	55.64	(± 11.72)	299.5
**^{13}C-LA**	linoleic acid (18:2n6)	20.93	(± 4.23)	297.5
**^{13}C-DHA**	docosahexaenoic acid (22:6n3)	0.25	(± 0.15)	349.5
AA	arachidonic acid (20:4n6)	0.96	(± 0.37)	
EPA	ecosapenatenoic acid (20:5n3)	0.10	(± 0.03)	
DH-γ-LNA	dihomo-γ-linolenic acid (20:3n6)	0.56	(± 0.23)	
αLNA	α linolenic acid (18:3n6)	0.90	(± 0.19)	

^{13}C-labelled FFA and un-labelled FFA (dissolved in 102 µL ethanol) were added to 200 mL perfusion medium containing 0.5% BSA under a stream of argon and incubated over night at 37°C prior each perfusion experiment. The labelled-DHA carried ^{13}C-isotopes on each C-atom position and the labelling efficiency was 97%-atoms (IsoLife, Wageningen, Netherlands). All other labelled-FFA carried also ^{13}C-isotopes on each C-atom position with a labelling efficiency of 99%-atoms (Sigma-Aldrich, Steinheim, Germany). FFA concentrations were determined by GC-MS and are expressed as mean (± SD) of 14 experiments. Masses (m-1) of ^{13}C-FFA determined by GC-MS in selected-ion monitoring (SIM).

Supplementary Table 2: Quality control parameters obtained during perfusion experiments.

Parameter	Mean	SD
Cotyledon (g)	25.4	(± 7.1)
Pressure (mbar)	48.7	(± 12.9)
Antipyrine FM ratio	0.55	(± 0.08)
pH fetal artery	7.48	(± 0.05)
pH maternal artery	7.57	(± 0.05)
Lactate fetal vein (mmoL/L)	1.00	(± 0.28)
Lactate maternal artery (mmoL/L)	4.61	(± 0.86)
Number of placentae	15	

Quality control parameters were determined every 30 min during the experiment and mean values (± SD) over 90 min were calculated for pressure, pH and lactate.

Supplementary Table 3: Characteristics of the study population.

	Lean		Obese		Statistics
Maternal age	32.0	(± 4.8)	30.0	(± 4.9)	n.s.
Pre-pregnancy BMI (kg/m²)	21.2	(± 1.8)	34.2	(± 5.3)	$p < 0.001$
Gestational age (weeks)	39.0	(± 0.4)	39.0	(± 0.4)	n.s.
Placental weight (g)	660	(± 142)	729	(± 143)	n.s.
Birth weight (g)	3367	(± 314)	3692	(± 686)	n.s.
Ponderal index (kg/m³)	26.4	(± 2.8)	28.6	(± 2.9)	n.s.
Fetal sex (n female/n male)	(6/2)		(3/4)		
Number of placentae	8		7		

Study subjects were classified according to maternal pre-pregnancy BMI into lean (BMI ≤ 25 kg/m²) or obese (BMI ≥ 30 kg/m²) group. Values are expressed as mean (± SD). Differences between lean and obese group were tested by the non-parametric Mann-Whitney U test. P-values < 0.05 were defined as statistical significant. n.s.: not significant.

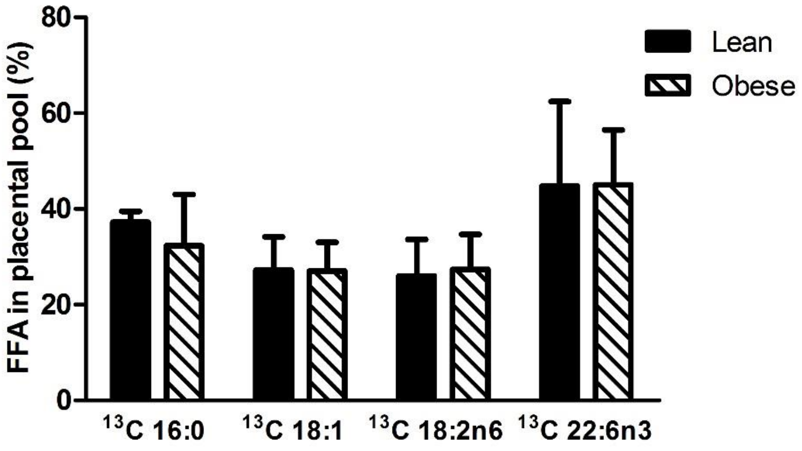

Supplementary Figure 1: FFA uptake in perfused tissue. ^{13}C-FFA concentration in maternal reservoir before and after, and in fetal outflow after 90 min perfusion experiment were determined by GC-MS. ^{13}C-FFA uptake in placental tissue (metabolic pool, mean ± SD) was calculated. The sum of ^{13}C-FFA absolute quantity in maternal and fetal reservoir (t 90 min) was subtracted from the absolute quantity in the maternal reservoir (before the experiment, t 0 min). Results are expressed as percentage of ^{13}C-FFA absolute quantity in maternal reservoir (t 0 min). Lean (n = 4), Obese (n = 3). Non-parametric group comparison (Mann Whitney U test) was performed.
